# A fiber-modified adenoviral vector interacts with immunoevasion molecules of the B7 family at the surface of murine leukemia cells derived from dormant tumors

**DOI:** 10.1186/1476-4598-10-105

**Published:** 2011-08-31

**Authors:** Elodie Grellier, Katia Lécolle, Sophie Rogée, Cyril Couturier, Jean-Claude D'Halluin, Saw-See Hong, Pascal Fender, Pierre Boulanger, Bruno Quesnel, Morvane Colin

**Affiliations:** 1 INSERM UMR 837, 1, rue Polonovski, 59045 Lille, France; 2 Univ. Lille Nord de France, 59000 Lille, France; 3 Institut de Recherches sur le Cancer de Lille, Place de Verdun, 59045 Lille, France; 4 Univ. Lille Nord de France, UDSL, Faculté de Médecine, IMPRT, JPARC, Place de Verdun, 59045 Lille, France; 5 INSERM UMR 761, Biostructures & Drug Discovery, Université de Lille 2, Institut Pasteur de Lille, Faculté de Pharmacie de Lille, 3, rue du Professeur Laguesse. Institut Pasteur de Lille, IFR 142 - PRIM, 59000 Lille, France; 6 Université Lyon 1 & UMR INRA-754, Rétrovirus & Pathologie Comparée, 50, avenue Tony Garnier, 69366 Lyon Cedex 07, France; 7 Unit of Virus Host Cell Interactions (UVHCI), UMI 3265 (CNRS/UJF/EMBL), 6, rue Jules Horowitz, 38042 Grenoble, France; 8 Service des Maladies du Sang, Centre Hospitalier et Universitaire de Lille, Rue Polonovski, 59000 Lille, France

**Keywords:** B7-H1, B7.1, dormant leukemia cells, adenovirus, adenovirus vector, atadenovirus, chimeric fiber

## Abstract

Tumor cells can escape the immune system by overexpressing molecules of the B7 family, e.g. B7-H1 (PD-L1 or CD86), which suppresses the anti-tumor T-cell responses through binding to the PD-1 receptor, and similarly for B7.1 (CD80), through binding to CTLA-4. Moreover, direct interactions between B7-H1 and B7.1 molecules are also likely to participate in the immunoevasion mechanism. In this study, we used a mouse model of tumor dormancy, DA1-3b leukemia cells. We previously showed that a minor population of DA1-3b cells persists in equilibrium with the immune system for long periods of time, and that the levels of surface expression of B7-H1 and B7.1 molecules correlates with the dormancy time. We found that leukemia cells DA1-3b/d365 cells, which derived from long-term dormant tumors and overexpressed B7-H1 and B7.1 molecules, were highly permissive to Ad5FB4, a human adenovirus serotype 5 (Ad5) vector pseudotyped with chimeric human-bovine fibers. Both B7-H1 and B7.1 were required for Ad5FB4-cell binding and entry, since (i) siRNA silencing of one or the other B7 gene transcript resulted in a net decrease in the cell binding and Ad5FB4-mediated transduction of DA1-3b/d365; and (ii) plasmid-directed expression of B7.1 and B7-H1 proteins conferred to Ad5FB4-refractory human cells a full permissiveness to this vector. Binding data and flow cytometry analysis suggested that B7.1 and B7-H1 molecules played different roles in Ad5FB4-mediated transduction of DA1-3b/d365, with B7.1 involved in cell attachment of Ad5FB4, and B7-H1 in Ad5FB4 internalization. BRET analysis showed that B7.1 and B7-H1 formed heterodimeric complexes at the cell surface, and that Ad5FB4 penton, the viral capsomere carrying the fiber projection, could negatively interfere with the formation of B7.1/B7-H1 heterodimers, or modify their conformation. As interactors of B7-H1/B7.1 molecules, Ad5FB4 particles and/or their penton capsomeres represent potential therapeutic agents targeting cancer cells that had developed immunoevasion mechanisms.

## Background

Tumor cells express numerous molecules at their surface that may influence their recognition by the immune system. Among them, proteins of the B7 family play important roles in the immunoevasion of tumor cells and can suppress T-cell-mediated immunity by binding to the inhibitory receptor CTLA-4, e.g. B7.1 (or CD80) and B7.2 (or CD86). Tumor cells that express B7.1 may be shielded from direct cytotoxic T-cell (CTL)-mediated killing [1-3]. Other members of the B7 family include B7-H1 (PD-L1 or CD274), B7-DC (PD-L2), ICOS-L, B7-H3 and B7-H4, but only B7-H1 and B7-H4 have been indisputably shown to play a role in the immunoevasion of cancer cells [4]. B7-H1 binds to its receptor PD-1, and this binding mediates immunosuppression [5]. B7-H1 also binds to B7.1 [6], but the function of this interaction remains unclear. B7-H1 suppresses the CTL-mediated killing of tumor cells, induces T-cell anergy and likely participates in T-cell exhaustion in cancer, as PD-1 is abundantly expressed on T-cells that infiltrate the tumor microenvironment. B7-H1 is constitutively expressed by several human tumors, and is induced when cancer cells are stimulated with interferon-γIFN-γ) and ligands of Toll-like receptors (TLR) [7-9]. Using a DA1-3b mouse model of tumor dormancy, we previously demonstrated that a minor population of dormant leukemia cells persists in equilibrium with the immune system for long periods of time. Dormant leukemia cells suppressed CTL-mediated killing by overexpressing B7-H1 and B7.1 [10-12]. All these observations suggested that the B7-H1 and B7.1 molecules of the B7-family could represent potential targets for new antitumor strategies (reviewed in [13]).

Cell surface molecules in cancer cells have been considered as privileged targets in cancer therapy, but mostly as targets of therapeutic monoclonal antibodies (mAb) [14]. Alternative therapeutic methods include the use of oncolytic viral vectors naturally directed, or genetically retargeted to specific molecules of the cell surface, capable of triggering tumor cell death. Recombinant oncolytic adenoviruses offer several advantages over other oncolytic viral vectors: (i) they have a large cloning capacity, (ii) are relatively easy to produce to high titers, with vector stocks remaining stable over a long period of storage, and (iii) their therapeutic effects do not require the viral DNA insertion into the host genome [15-18]. However, with the exception of certain members of species B adenoviruses, e.g. HAdV3, which have the natural ability to bind to B7.1 and B7.2 [19] and to efficiently transduce B7.1- and B7.2-expressing malignant glioma cells [20], the usage of adenoviruses in cancer gene therapy is limited, due to the low level (or absence) of expression of high affinity receptor for adenoviruses in cancer cells, or/and their poor accessibility at the cell surface. This is the case for the Coxsackie and Adenovirus human Receptor (hCAR), one of the natural receptors for adenoviral species A, C, D, E and F, which is located in the tight junctions and expressed at low levels in cancer cells [21], and for desmoglein-2, the receptor of HAdV3, also found in tight junctions [22]. Different strategies of adenoviral vectors have been proposed, and the most popular consisted of fiber capsomere modifications, to allow the vectors to attach to newly defined cell targets for efficient virus entry [15,17,18].

A fiber-modified, ß-galactosidase (ß-gal)-expressing adenoviral vector, originally called HAdV5-F2/BAdV4-ßgal and abbreviated Ad5FB4 in the present study, was previously constructed and characterized. Ad5FB4 is a human adenovirus serotype 5 which carries chimeric, human/bovine fibers [23-27]. It does not recognize the ubiquitous hCAR, and binds to cells *via *an attachment receptor different from the heparan sulfate proteoglycans [25]. Transduction of hCAR-negative and hCAR-positive cells occurs *via *a clathrin-independent endocytic pathway involving lipid raft/caveolae [27]. An advantage of the altered tropism of the Ad5FB4 vector is the restriction of its infection repertoire of cells, which therefore limits the vector dissemination [24]. This results in the reduction of adenovirus-associated humoral and innate cytokine immune responses upon intravenous administration of Ad5FB4 vector to mice [26].

In the present study, we tested Ad5FB4 on malignant cells refractory to conventional Ad5-based vectors, and found that the permissiveness of murine leukemia cells to Ad5FB4 correlated with their dormancy time and the expression level of B7.1 and B7-H1 molecules at their surface. Results from *in vitro *and *in vivo *experiments suggested that B7.1 and B7-H1 molecules played different roles in Ad5FB4-mediated transduction of murine dormant leukemia cells, with B7.1 involved in cell attachment of Ad5FB4, and B7-H1 in its cellular uptake. Our data also suggested that the interaction between B7.1 and Ad5FB4 was mediated by the penton capsomeres (or penton base-linked fibers) of the vector capsid. *In situ *BRET analysis showed that B7.1 interacted with B7-H1 to form heterodimers at the cell surface, and that Ad5FB4 penton capsomeres interfered negatively with the formation of these complexes. Our finding that tumor cell surface molecules of the B7 family implicated in immunoevasion mechanisms were recognised by the adenoviral vector Ad5FB4 offered novel opportunities for cancer therapy, using intrinsically B7-targeted Ad5FB4 vectors for therapeutic gene transfer. Alternatively, Ad5FB4 penton capsomeres, via their negative interference with the B7-H1/B7.1 heterodimer formation, might be used as therapeutic agents to decrease the amounts of these complexes at the tumor cell surface, and hence lower their capacity to resist to anti-tumor T-cell responses.

## Methods

### Plasmids

Mouse B7-H1 cDNA was kindly provided by M. Azuma [28], and pSelect-B7.1 was purchased from Invivogen (San Diego, CA). For fusion constructs, the stop codons of the murine coding sequences of B7-H1 and B7.1 were removed from the plasmids. PCR products were cloned in phase with either Rluc8 or YPet into the pcDNA3 vector [29].

### Cell culture and transfection

Dormant leukemia cells (DA1-3b/d35, DA1-3b/d90 and DA1-3b/d365) were established as described previously [12]. DA1-3b and DA1-3b-derived cell lines, Raji and Jurkat cells were cultured in RPMI-1640 medium with 10% fetal bovine serum (FBS), 1% non-essential amino acids (NEAA), and 1 mM sodium pyruvate. Epithelial cells HEK-293 and A549 were cultured in DMEM medium supplemented with 10% FBS and 1% NEAA. HeLa cells were grown in DMEM supplemented with 10% FBS, 4.5 g/L glucose, and 1 mM glutamine. Transfections for establishing transient expression were performed using Fugene6 HD (Roche, Meylan, France). Cells were maintained in a humidified incubator at 37°C with 5% CO_2_.

### Antibodies and immunodetection of cell surface molecules. (i) B7-H1/B7.1

Cells (10^6^-aliquots) were first incubated with 5 μg/mL of Fc-receptor blocking antibody (rat anti-mouse CD16/CD32; BD Biosciences, Le Pont-De-Claix, France) for 5 min at 4°C. Cells were then reacted with monoclonal antibodies against PE-labeled mouse/human B7-H1, FITC-labeled mouse/human B7.1 (eBioscience, San Diego, CA; http://www.eBioscience.com), and the corresponding control isotype, at 40 μg/mL and 4°C for 1 h. Cell surface expression of B7-H1 or B7.1 was quantitated by flow cytometry, using an EPICS XL MLC Coulter flow cytometer. For detection of cell surface molecules B7.1 and B7-H1 after adenoviral vector cellular uptake, aliquots of DA1-3b/d365 cells in suspension (1.5 × 10^6^) were incubated at 4°C for 90 min with Ad5FB4 vector doses of 5,000 or 10,000 physical particles per cell (vp/cell) in serum-free medium. Cells were then transferred to 37°C for 10 min to allow for vector internalization, and probed for B7-H1 and B7.1 by flow cytometry, as described above. ***(ii) CAR*. **CAR was detected by flow cytometry, using polyclonal antibody against mouse CAR (Santa Cruz Biotechnology, Cat.#sc-10313), or the corresponding control isotype (goat IgG; Santa Cruz biotechnology, Cat#sc-3887). (iii) ***IgFcR***. Cell surface expression of immunoglobulin Fc receptors was assessed by flow cytometry, using rat anti-mouse CD16/CD32 (Fc receptor blocking antibody; BD Biosciences, Le Pont-De-Claix, France) or the corresponding control isotype, and Alexa fluor^® ^488-labeled goat anti-rat IgG (Invitrogen, A11006), and quantitated by flow cytometry, using an EPICS XL MLC Coulter flow cytometer.

### Cell sorting

DA1-3b cells (10^7^) were suspended in 3 mL of ice-cold PBS containing 1% BSA and 2 mM EDTA, and incubated with monoclonal PE-labeled antibody against B7-H1 or control, irrelevant isotopic antibody, as described above. DA1-3b cells expressing B7-H1 at low and high levels, respectively, were sorted using an Epics Altra Coulter cell sorter.

### B7-H1/B7.1 siRNA knockdown

DA1-3b/d365 cells were transfected by electroporation with siRNA (Thermo Fisher, Dharmacon technology, Belgium) using 3.3 nmol/cell of siRNA against murine B7-H1 (5'-CACAAUUCgAggAgACgUAUU-3') or B7.1 (5'-gAAUUACUggCAUCAAUA-3'). Negative control siRNA consisted of scramble sequences. B7-H1 or B7.1 expression was immunodetected as described above, and the silencing effect determined at 24, 48, 96 and 144 h after electroporation.

### Adenovirus vector amplification and labeling

The genetic constructions of the E1-deleted adenoviral vectors Ad5 and Ad5FB4 containing the ß-gal reporter gene have been described previously [23,24]. In the chimeric Ad5FB4 fiber, the junction between human serotype 2 fiber (F2) and bovine serotype 4 (BAdV4) fiber was situated in the shaft repeat 7 at the GKL (glycine-lysine-leucine) motif, to generate the chimeric fiber F2/BAdV4 [24], abbreviated FB4 in the present study. The Ad5FB4 and Ad5 vectors were amplified and purified following conventional protocols. Since Ad5FB4 had a lower tropism for epithelial cells, compared to Ad5, it was not possible to compare their infectious titers by conventional plaque assays on HEK-293 cell monolayers. Stocks of purified vectors were titrated by optical measurement of the viral DNA concentration at 260 nm, and the vector titer expressed as vp/mL. Fluorescent labeling of vector particles with carboxyfluorescein succinimidyl ester (FAM; Invitrogen, Cergy-Pontoise, France) was performed as previously described [27].

### Adenovirus infection

Ad5FB4 and Ad5 infections were carried out as previously described [27], except for the virus inoculum which was eliminated by low-speed centrifugation of the infected cells (800 × *g*, 5 min) at 24 h post-infection (pi). Cells were then resuspended in culture medium with 4% FBS and maintained for an extra 48 h for murine cells, or an extra 24 h for human cells. The ß-gal activity was determined using fluorescein-ß-D-galactopyranoside or the colorimetric X-gal staining procedure (Fisher scientific, Belgium), as previously described [24].

### Vector-cell binding and internalization

Cell aliquots (1.5 × 10^6^) were incubated at 4°C for 1 h in suspension with FAM-labeled Ad5 or FAM-labeled Ad5FB4 at 10^11 ^vp/mL in serum-free medium. Cells were rinsed with PBS containing 1% BSA (PBS-BSA), and cell-bound vector particles were quantitated using flow cytometry (FACS). For internalization assays, cells and vector were incubated at 4°C for 1 h, then transferred to 37°C and further incubated at this temperature for different periods of time, ranging from 5 min to 2 h. Before FACS analysis, cell samples were incubated for 15 min at 37°C with trypsin at 0.25% in 1 mM EDTA to detach vector particles possibly sequestered at the cell surface [30]. Cells were resuspended in PBS-BSA, and the amounts of internalized vector were quantitated by FACS analysis.

### Surface plasmon resonance (SPR)

SPR analyses were carried out using a BIAcore 2000. Recombinant mouse B7-H1, B7.1 and PD-1 were covalently immobilized onto separate flow cells of a CM5 biosensor chip by amine coupling according to the manufacturer's instructions. As a reference, another flow-cell surface was activated and deactivated. Protein samples were diluted in HBS (0.01 M Hepes, pH 7.4; 0.15 M NaCl; 0.005% P20), and the binding analyses were performed at 25°C with HBS as running buffer. A flow rate of 10 μL/min was used to inject B7-H1, B7-H2, B7.1 (R&D Systems Europe, Lille, France) and 20 μL/min to inject viral proteins; all samples were run five times. For vector particles analysis, SPR experiments (BIAcore 3000) were run on a CM4 sensorship at 5 μL/min using HBS-N (GE-Healthcare) supplemented with 2 mM CaCl_2. _Immobilisation of both B7.1 and B7-H1 was performed by interaction of these ligands diluted at 1 μg/mL in 10 mM sodium acetate buffer pH 4.2 on EDC-NHS activated flow-cells for 10 min at room temperature. After ethanolamine deactivation, vector particles were injected (1.10^10 ^vp in 25 μL of running buffer) and the signal from the ligand flow-cells was automatically subtracted from the background of an ethanolamine deactivated EDC-NHS flow-cell.

### Human B7.1, B7-H1 and PD-1 proteins and adenoviral capsid proteins

Recombinant B7-1-Fc, B7-H1-Fc, and PD-1-Fc fusion proteins used in SPR analyses were purchased from R&D Systems. Adenovirus penton protein (penton base-linked fiber) was isolated from adenovirus-infected 293 cell lysates, according to a conventional protocol adapted to fast protein liquid chromatography [31-34].

### BRET

At 24 h before transfection, cells (2 × 10^5^-aliquots) were plated in 6-well plates and transfected with increasing amounts of B7.1-YPet-, B7-H1-YPet- or IR-YPet-expressing plasmids (10 to 500 ng/well), and constant amounts (10 ng-aliquots) of plasmid expressing B7-H1-Rluc8 or B7.1-Rluc8 fusion protein. 48 h later, cells were collected and washed twice with PBS, and aliquots were placed in 384-well plates. Coelenterazine H substrate was added at a final molarity of 5 μM, and BRET was measured immediately. To analyze the effect of the penton, cells were incubated for 5 min without or with penton protein solution at 0.33, 0.66 or 132 ng/μL, followed by Coelenterazine H addition and BRET measurement. BRET was monitored using a lumino/fluorometer (Mithras; Berthold Technologies, France), allowing for the sequential integration of luminescence with two filter settings (Rluc filter, 485 ± 10 nm; YFP filter, 530 ± 12.5 nm). The emission signal values obtained at 530 nm were divided by the emission signal values obtained at 485 nm. The BRET ratio was calculated by dividing the emission signal value obtained with coexpressed donor and acceptor by that obtained with the donor protein expressed alone. Data from at least three independent experiments were averaged, and results expressed as milliBRET (mBRET), corresponding to the BRET ratio multiplied by 1,000. Donor saturation curves were determined as previously described [35,36].

### Statistical analyses

Data were presented as the mean of triplicate experiments (m ± SEM), and were representative of the results obtained from three independent experiments that produced similar results. Statistical analyses were performed using the Mann-Whitney test.

## Results

### Cell and vector nomenclature

Murine dormant leukemia cell lines have been previously established from long-term persistent tumor cells isolated from mice in a state of tumor dormancy [12,13]. In brief, C3H/HeJ mice have been immunized with irradiated, interleukin-12-treated or CD154-transduced DA1-3b cells, challenged with parental DA1-3b cells, and randomly sacrificed during a one-year follow-up period at day 35, 90 and 365, respectively. Dormant leukemia cells were collected from the spleen at these different times, and the amount of BCR/ABL mRNA quantitatively assayed using real-time PCR, leading to the following cell lines: DA1-3b/d35, DA1-3b/d90 and DA1-3b/d365 [12]. The ß-gal-expressing, fiber-modified adenoviral vector HAdV5-F2/BAdV4-ß-gal comprises of the human Ad5 DNA backbone containing a chimeric fiber gene. The vector particle consisted of serotype 5 capsid carrying chimeric fibers, each formed by the human serotype 2 fiber tail fused to the shaft and knob domains of bovine serotype 4 (BAdV4) [23-27]. For reasons of simplification, this human/bovine chimeric fiber vector was referred to as Ad5FB4 in the present study, and the control, ß-gal-expressing Ad5 vector with homotypic serotype 5 capsid proteins was abbreviated Ad5.

### Permissiveness of dormant leukemia cells to the chimeric fiber vector Ad5FB4

**Figure 1:**
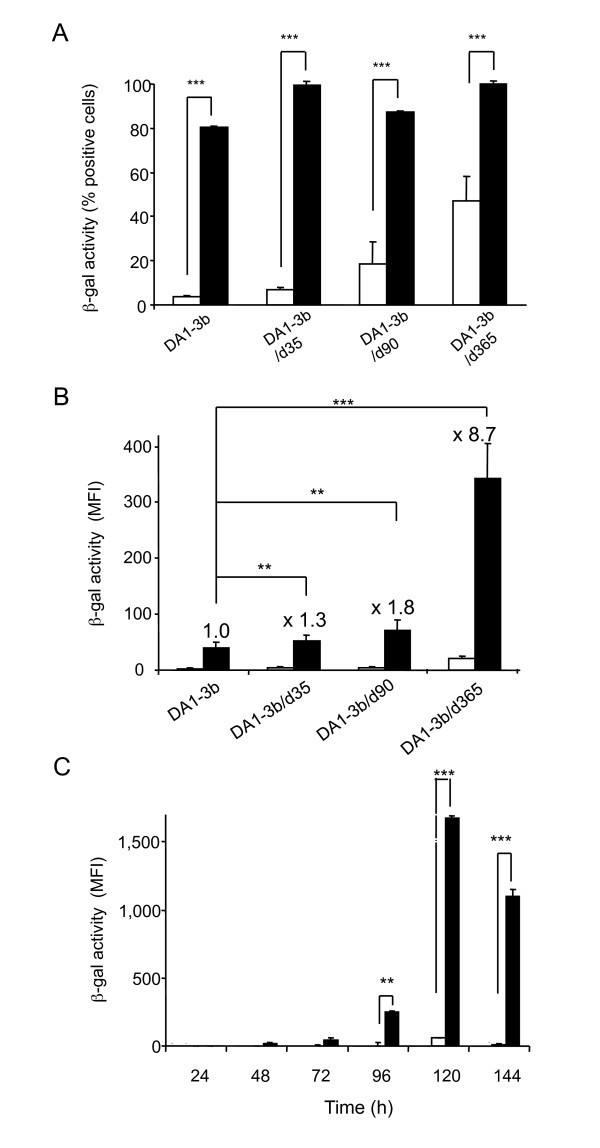
**Correlation between the efficiency of Ad5FB4-mediated transduction of murine dormant leukemia cells and the time of tumor dormancy**. **(A, B)**, DA1-3b cells and the DA1-3b-derived cell lines DA1-3b/35, DA1-3b/d90 and DA1-3b/d365 were incubated with ß-gal-expressing Ad5 (open square) or Ad5FB4 vector (black square) at 10^4 ^vp/cell for 2 h at 37°C in serum-free medium. The ß-gal activity was evaluated by flow cytometry at 72 h pi, and expressed as **(A) **the percentage of ß-gal-positive cells, or **(B) **mean fluorescence intensity (MFI). In (**B**), the numbers on top of the bars represented the fold enhancement of ß-gal activity relative to the value in DA1-3b cells, which was attributed the 1-value. **(C)**, DA1-3b/d365 cells were incubated with Ad5-ßgal (open square) or Ad5FB4-ßgal (black square) at 10^4 ^vp/cell for 2 h at 37°C, harvested at different times pi as indicated on the *x*-axis, and assayed for ß-gal expression. The ß-gal activity was expressed as MFI. Symbols: **, p < 0.01; ***, p < 0.001.

**Figure 2:**
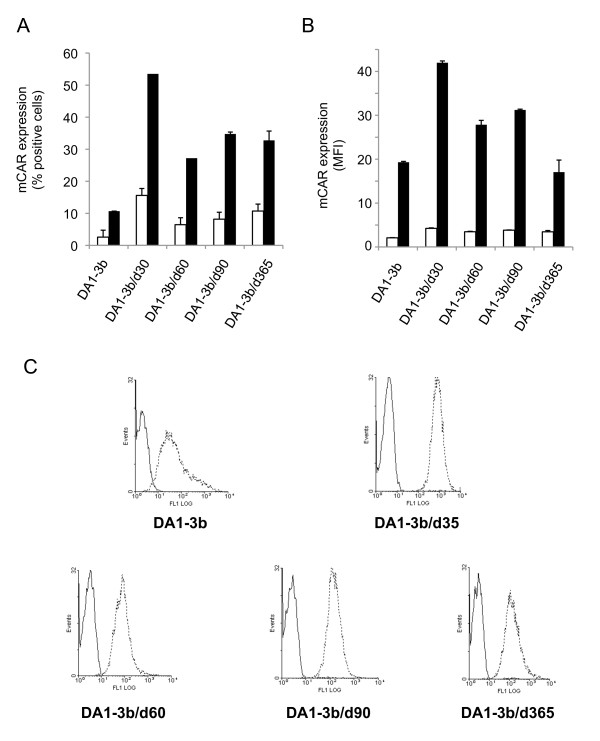
**Cell surface expression of mouse CAR (mCAR) and immunoglobulin Fc receptors in DA1-3b and DA1-3b-derived cell lines**. **(A, B)**, ***CAR expression***. Cells first incubated with Fc receptor blocking antibody (rat anti-mouse CD16/CD32), or the corresponding control isotype, were reacted with goat polyclonal antibody against mCAR followed by FITC-labeled secondary antibody (donkey anti-goat), and analyzed by flow cytometry. Results are expressed as (**A**) the percentage of mCAR-positive cells, and (**B**) MFI. The open bars in panels A and B correspond to the isotype-matched control antibodies. **(C)**, ***Fc receptors***. Cells incubated with Fc receptor blocking antibody (dotted line), or the corresponding control isotype (solid line), as above, were reacted with Alexa fluor^® ^488-labeled goat anti-rat IgG. Results are presented as conventional flow cytometry patterns.

The three cell lines DA1-3b/d35, DA1-3b/d90 and DA1-3b/d365 were incubated with equal physical particle inputs of the chimeric vector Ad5FB4 or control vector Ad5. The efficiency of cell transduction was assayed by the percentage of ß-gal-positive cells and the level of ß-gal expression, determined by the mean fluorescence intensity (MFI) using a fluorescent ß-gal substrate. We found that Ad5FB4 transduced murine dormant leukemia cells with a higher efficiency, compared to Ad5 vector (Figure 1A). Interestingly, the percentage of ß-gal-positive cells was not not significantly different for the various DA1-3b cell lines derived from *in vivo *passages. Rather, the level of Ad5FB4-mediated transgene expression correlated with increased dormancy: the lowest ß-gal activity was observed in DA1-3b/d35, the highest in DA1-3b/d365 cells, with an intermediate value in DA1-3b/d90 cells (Figure 1B). Of note, Ad5 transduction, as determined by the percentage of transduced cells, also correlated with the length of *in vivo *passage (Figure 1A). Interestingly, the transgene expression progressively increased with the period of time after transduction, as exemplified with Ad5FB4-transduced DA1-3b/d365 cells, which showed a 30-fold enhancement of ß-gal activity between 72 and 120 h posttransduction (Figure 1C). These results suggested that the DA1-3b/d365 cells provided a more favorable environment for the expression of the transgene transduced by Ad5FB4 or Ad5. However, the possibility of an increased expression of mouse CAR (mCAR) in the different cell lines, and/or modifications of intracellular factors, e.g. transcription factors, was envisaged. All cell lines were found to express mCAR at their surface at various levels, as assayed by flow cytometry (Figure 2A, B). The pattern of mean fluorescence intensity in the different lines roughly paralleled that of the percentage of mCAR-positive cells, and both data clearly showed no direct correlation between mCAR levels, dormancy time and increased Ad5 (or Ad5FB4) transduction.

### Correlation between cell surface levels of B7-H1 and B7.1 molecules and cell permissiveness to Ad5FB4

**Figure 3:**
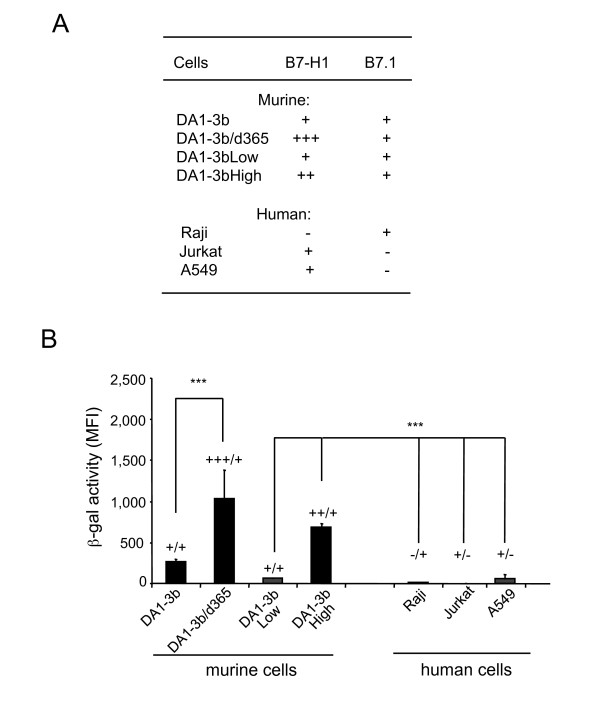
**Permissiveness to Ad5FB4 and expression of B7 molecules at the surface of murine and human cells**. **(A)**, Cells were reacted with PE-labeled antibody against murine or human B7-H1 or B7.1, and analyzed by flow cytometry. Symbols (+++), (++), (+), and (-) refer to the respective levels of surface expression of B7-H1 and B7.1, evaluated by MFI, and expressed as arbitrary units (AU): (-), undetectable; (+), 10-20; (++), 50-80; (+++), 200-300 AU. **(B)**, Cells were transduced by ß-gal-expressing Ad5FB4 vector at 10^4 ^vp/cell, and ß-gal assayed at 72 h pi in murine cells, and at 48 h pi in human cells. Symbols: ***, p < 0.001; (+++/+), (++/+), (+/+), (+/-) and (-/+) refer to the respective levels of surface expression of B7-H1/B7.1, as evaluated in panel (A).

The apparent correlation between the level of Ad5FB4-mediated ß-gal expression and the dormancy time, as shown in Figure 1B, suggested that the degree of permissiveness of DA1-3b cells to Ad5FB4 depended on the level of expression of B7-H1 and B7.1 molecules at the cell surface. To test this hypothesis, we analyzed the permissiveness to Ad5FB4 of various mouse and human cell lines differing by their absolute and relative levels of surface-expressed B7-H1 and B7.1 (Figure 3A). We found that the Ad5FB4-mediated cell transduction required both B7.1 and B7-H1 molecules, but the transduction efficiency seemed to correlate with the B7-H1 levels (Figure 3B).

Since intrinsic properties of individual cell lines could influence the cell transduction levels independently of the B7.1/B7-H1 pattern, we sorted the DA1-3b cell line into two subpopulations, on the basis of their levels of surface expression of the B7-H1 protein. The two subclones isolated, referred to as DA1-3bLow and DA1-3bHigh, respectively (Figure 3A), were assayed for ß-gal activity after Ad5FB4 transduction. The results indicated that the subpopulation of high B7-H1-expressors was transduced with a 7-fold higher efficiency, compared to the subpopulation of low B7-H1-expressors (Figure 3B). This confirmed that the transduction efficiency by Ad5FB4 correlated with the level of expression of the B7-H1 protein and the ratio of B7-H1 to B7.1. Interestingly, the fact that both the original DA1-3b and the DA1-3bLow subclone were low expressors of B7-H1 and B7.1 but exhibited marked differences in ß-gal expression (Figure 3B) suggested the existence of a threshold of B7-H1 at which the influence on ß-gal expression became apparent. This threshold was consistent with a cooperative effect between B7-H1 and B7.1 for Ad5FB4 uptake, with B7-H1 being the limiting factor: a certain concentration of B7-H1 molecules would be required to form B7.1-B7-H1 complexes at the cell surface and mediate the cell entry of the vector, as described below.

A similar correlation between permissiveness to Ad5FB4 and B7.1/B7-H1 pattern was observed for human cell lines Raji, Jurkat and A549 (Figure 3A, B). Unfortunately, no control data could be provided using mouse cell lines expressing only one or the other B7 molecule, since tumor dormancy was consistently associated with the expression of both B7 products at the cell surface. In the case of human cells however, we found no cell line that coexpresses B7-H1 and B7.1. This was the reason why indirect methods, e.g. specific RNA interference and single or double expression of B7 molecules via plasmid transfection, were used (Cf. below).

### Respective roles of B7.1 and B7-H1 molecules in the cellular uptake of Ad5FB4

To determine whether B7.1 or/and B7-H1 could be used by Ad5FB4 as receptors for attachment and/or entry into murine cells, the method of choice would consist of cell binding competition experiments between the vector and (i) the soluble form of the candidate receptor(s), and between the vector and (ii) the soluble ligands of the candidate receptor(s). However, the recombinant B7 molecules available commercially consisted of the B7.1 and B7-H1 ectodomains fused to the immunoglobulin (Ig) Fc domain (B7.1-Fc and B7-H1-Fc), and the same was true for the natural ligand of B7-H1, Programmed Death-1 (PD-1; [37]). Since the binding of the Fc-fused proteins to Fc receptors present at the surface of all DA1-3b cells (Figure 2B) would introduce a bias in the competition assays with B7.1-Fc, B7-H1-Fc and PD-1-Fc, we used alternative methods to assess the respective roles of B7.1 and B7-H1 proteins in the cell permissiveness to Ad5FB4.

#### (i) B7.1 knockdown and Ad5FB4-cell binding

**Figure 4:**
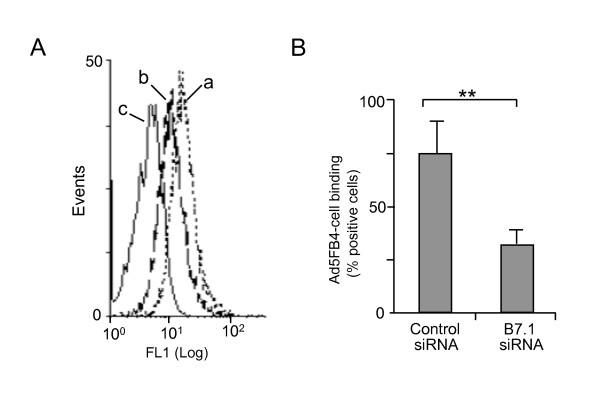
**Effect of B7.1 silencing on the Ad5FB4-DA1-3b/d365 cell binding**. Cells were electroporated with control siRNA or siRNA against B7.1 (3 nmol/cell), maintained in culture for 96 h, transferred to 4°C, then incubated with FAM-labeled Ad5FB4 at 10^4 ^vp/cell for 90 min at 4°C. **(A)**, The efficiency of B7.1 silencing was verified by flow cytometry, using PE-labeled antibody against murine B7.1. (**a**), control siRNA; (**b**), siRNA against B7.1; (**c**), control irrelevant isotypic antibody. **(B)**, Cell-bound Ad5FB4 vector particles were quantitated by flow cytometry analysis of cell surface-associated fluorescent signal, and results expressed as the percentage of positive, fluorescent cells.

B7.1 gene silencing was performed in DA1-3b/d365 cells, using small interfering RNA (siRNA). The reduction of surface expression of B7.1 was found to be maximal at 96 h after siRNA electroporation, as assessed by flow cytometry (Figure 4A). DA1-3b/d365 cells electroporated with siRNA were then transferred to 4°C, and incubated with aliquots of FAM-labeled, fluorescent Ad5FB4 particles for 90 min at 4°C. Cell-bound Ad5FB4 particles were quantitated by flow cytometry analysis of the fluorescent signal. A significant reduction (ca. 50%) in cell binding was observed in B7.1-siRNA-treated cells, compared to control siRNA-treated cells (Figure 4B). This suggested that B7.1 played the role of attachment receptor for Ad5FB4 at the surface of DA1-3b/d365 cells.

#### (ii) B7-H1 knockdown and cellular internalization of Ad5FB4

**Figure 5:**
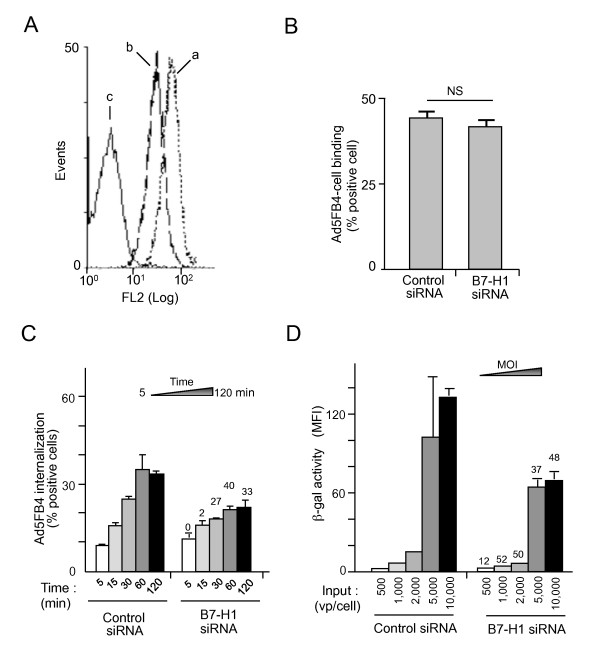
**Effect of B7-H1 silencing on cell binding and uptake of Ad5FB4 by DA1-3b/d365 cells**. **(A, B), *Ad5FB4-cell binding***. Cells were transfected with control siRNA or siRNA against B7-H1 (3 nmol/cell), and maintained in culture for 144 h. They were then incubated with FAM-labeled Ad5FB4 at 10^4 ^vp/cell for 90 min at 4°C. **(A)**, The efficiency of B7-H1 silencing was verified by flow cytometry analysis of siRNA-treated cells, using PE-labeled antibody against murine B7-H1. Curves were: **a**, control siRNA; **b**, siRNA against B7-H1; **c**, control irrelevant isotypic antibody. **(B)**, Cell-bound Ad5FB4 vector particles were quantitated by flow cytometry, and results expressed as the percentage of positive, fluorescent cells. **(C, D)**, ***Cellular uptake and transduction***. Cells were transfected with control siRNA or siRNA to B7-H1 (3 nmol/cell) and maintained in culture for 144 h. They were incubated with FAM-labeled Ad5FB4 at 10^4 ^vp/cell for 90 min at 4°C, followed by transfer to 37°C for different periods of time, ranging from 5 to 120 min. **(C**), ***Cellular internalization of Ad5FB4 particles***. Intracellular vector was quantitated by flow cytometry, and results expressed as the percentage of positive, fluorescent cells. The numbers on top of the rightmost series of bars represented the percentage of decrease in the number of positive cells, relative to that number in control siRNA-treated cell samples at time 0 of transfer to 37°C, which was attributed the 100%-value. **(D**), ***Ad5FB4-mediated DA1-3b/d365 cell transduction***. B7-H1 siRNA-treated cells were taken at 144 h after transfection and incubated with the Ad5FB4 vector for 2 h at 37°C at increasing vector doses, ranging from 5 × 10^2 ^to 10^4 ^vp/cell. After an additional 72 h at 37°C, ß-gal activity was assayed in cell lysates, and expressed as MFI. The numbers on top of the rightmost series of bars represented the decrease of ß-gal activity (as a percentage of control), relative to the value in control siRNA-treated cells infected with 5 × 10^2 ^vp/cell, which was attributed the 100%-value. MOI, multiplicity of infection, expressed as vp/cell.

Specific siRNA was also used to decrease the surface expression of B7-H1 (Figure 5A). After B7-H1 silencing, there was no detectable change in the binding of FAM-labeled Ad5FB4 to DA1-3b/d365 cells at low temperature (Figure 5B). For cell internalization assays, fluorescent-labeled Ad5FB4 particles were incubated with DA1-3b/d365 cells at 4°C to allow for vector-cell attachment, then samples transferred to 37°C. Vector particles remaining trapped at the cell surface were removed by digestion with trypsin [30], and the amounts of fluorescent-labeled Ad5FB4 particles internalized by DA1-3b/d365 cells were measured by flow cytometry at different times posttransfer to 37°C (5 to 120 min). The cell transduction efficiency, measured by the ß-gal activity, was also determined at vector doses, ranging from 500 to 10,000 vp/cell. The cellular internalization of Ad5FB4 was significantly reduced in B7-H1-silenced DA1-3b/d365 cells at all time points, with a maximum 40% inhibition at 60 min (Figure 5C). Likewise, the transduction efficiency significantly decreased after B7-H1 knockdown at all vector doses used: a maximum 50% inhibition was observed at 1,000 to 2,000 vp/cell, but the inhibitory effect almost plateaued at higher vector doses (Figure 5D).

#### (iii) B7-mediated gain of viral receptor function by Ad5FB4-refractory cells

**Figure 6:**
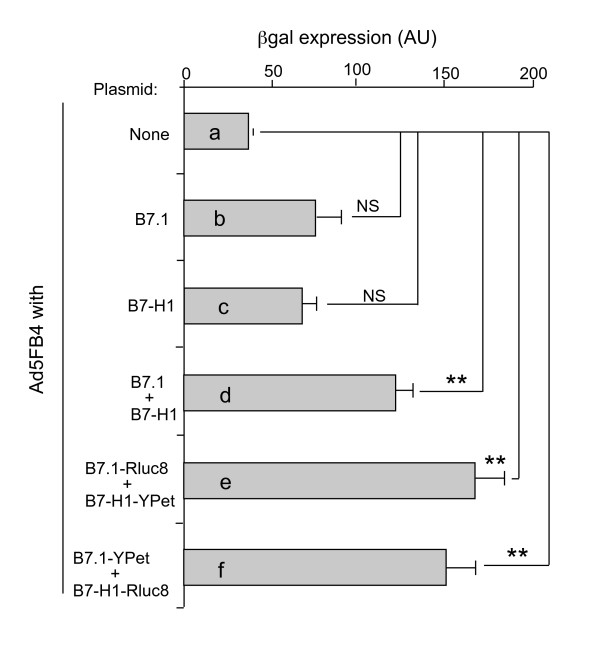
**B7-mediated gain of Ad5FB4-permissiveness by HeLa cells**. HeLa cells were transfected with B7.1- and B7-H1-expressing plasmids prior to transduction by Ad5FB4 at 500 vp/cell. Cells were fixed and stained with X-gal substrate at 48 h after incubation with Ad5FB4, and ß-gal activity (expressed as arbitrary units; AU) was determined by light microscopy, using the Image J software (*n *= 4 per condition). **(a)**, Control, Ad5FB4-transduced, nontransfected cells. **(b)**, Ad5FB4-transduced cells expressing B7.1 alone. **(c)**, Ad5FB4-transduced cells expressing B7-H1 alone. **(d)**, Ad5FB4-transduced cells coexpressing B7.1 and B7-H1. **(e)**, Ad5FB4-transduced cells coexpressing B7.1-Rluc8 and B7-H1-YPet fusion proteins. **(f)**, Ad5FB4-transduced cells coexpressing B7-H1-Rluc8 and B7.1-YPet. Symbols: **, p < 0.01; NS, non significant.

We have shown in previous studies that HeLa cells have a low permissiveness to Ad5FB4 infection [23-25,27]. Transfection of HeLa cells with one single plasmid expressing B7.1 or B7-H1 protein alone did not show any significant increase in their permissivity to Ad5FB4 (Figure 6, compare samples a, b and c). Of note, the relatively high background of ß-gal expression in control, nontransfected cells (Figure 6a) was due to the fact that HeLa cells are not totally refractory to Ad5FB4, as already reported [23-25,27]. However, coexpression of B7.1 and B7-H1 proteins by double transfection conferred to HeLa cells a full permissiveness to Ad5FB4 (Figure 6d). Taken together, these results suggested that the uptake of Ad5FB4 by HeLa cells and murine leukemia cells DA1-3b/d365 required the coexpression of B7-H1 and B7.1 molecules at the cell surface. In addition, the data with DA1-3b/d365 cells suggested that B7-H1 and B7.1 were involved in two different mechanisms: B7.1 would be mainly responsible for cell attachment of vector particles, while B7-H1 would contribute to their internalization, and to the efficiency of vector-mediated transduction.

### In vitro interactions between B7.1, B7-H1, Ad5FB4, and adenoviral capsid components

**Figure 7:**
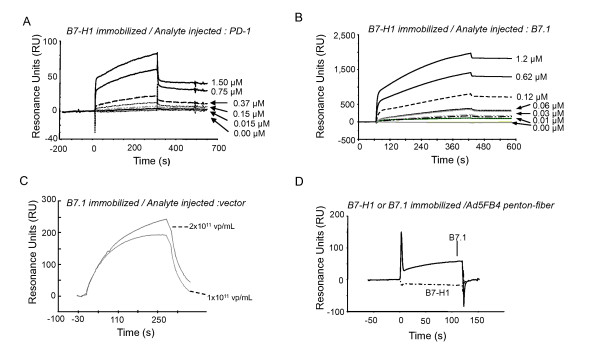
**SPR analysis of *in vitro *interactions between B7.1, B7-H1 and Ad5FB4**. **(A)**, Interaction of surface-immobilized B7-H1 with **(A) **PD-1, or **(B) **B7.1, injected at different molarities. **(C)**, Immobilized B7.1 was reacted with Ad5FB4 vector particles, injected at 1 × 10^11 ^or 2 × 10^11 ^vp/mL. **(D)**, Immobilized B7.1 (solid line) or B7-H1 (dotted line) proteins were reacted with Ad5FB4 penton capsomeres. Results are expressed as resonance units (RU).

The possible interactions between the different recombinant proteins B7-H1-Fc, B7.1-Fc and PD-1-Fc on one hand, and between B7-H1-Fc or B7.1-Fc and Ad5FB4 vector on the other hand, were investigated using surface plasmon resonance (SPR). SPR analysis confirmed the interaction between B7-H1 and PD-1 already reported [5]: the value for the *K*_D _(0.82 μM; Figure 7A) was in good consistency with that previously determined (*K*_D _= 0.77 μM; [5]). Our sensorgrams also confirmed the interaction between B7-H1 and B7.1 recently described [6]: the B7-H1-B7.1 binding reaction was found to occur with *K*_D _values of 0.38 μM B7.1 or 1.07 μM B7-H1, when B7.1 and B7-H1 were alternatively used as ligand or ligate (Figure 7B).

SPR analysis, using immobilized B7.1-Fc and B7-H1-Fc proteins, showed that Ad5FB4 vector particles interacted directly with B7.1-Fc (Figure 7C), but not with B7-H1-Fc (not shown). Purified penton protein, the adenoviral capsomere responsible for cell attachment and endocytosis (reviewed in [17,18]), and consisting of penton base-linked fiber, was isolated from Ad5FB4-infected HEK-293 cells as free, nonencapsidated viral protein [31,33]. Samples of Ad5FB4 penton protein were assayed by SPR on immobilized B7.1-Fc and B7-H1-Fc proteins. Ad5FB4 penton interacted with B7.1-Fc, but not with B7-H1-Fc (Figure 7D), suggesting that the binding of Ad5FB4 particles to cell surface-displayed B7.1 molecules occurred via their fiber apical projections [17,18].

Interestingly, when Ad5FB4 particles were preincubated with the B7.1-Fc protein prior to injection onto surface-immobilized B7-H1-Fc, no extra signal over the basic signal of B7.1-B7-H1 interaction was detected on the sensorgrams (data not shown), suggesting that the binding of Ad5FB4 to B7.1 did not increase the affinity of B7.1 to B7-H1. The next experiments were designed to further explore the respective roles of B7.1 and B7-H1 in the cell binding and entry of Ad5FB4 in the context of the DA1-3b/d365 plasma membrane.

### Cellular internalization of B7-H1 molecules upon Ad5FB4 uptake

**Figure 8:**
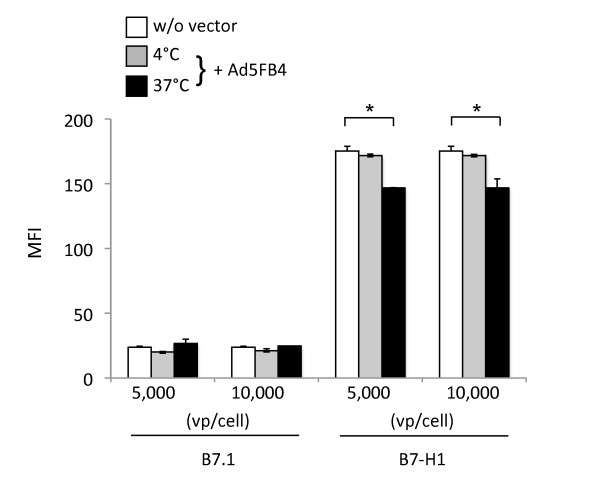
**Cell surface status of B7 molecules upon Ad5FB4 internalization**. DA1-3b/d365 cells in suspension were incubated for 90 min at 4°C without (open bars) or with Ad5FB4 vector (filled bars) at 5,000 or 10,000 vp/cell. Cells were transferred to 37°C to allow for Ad5FB4 endocytosis and entry. The presence of B7-H1 and B7.1 molecules at the cell surface was assessed by flow cytometry in control cells (open bars), in cells taken after vector attachment at 4°C (grey bars), and in cells taken after 10 min at 37°C (black bars). Results shown are MFI. Symbols: *, p < 0.05.

DA1-3b/d365 cells in suspension were incubated with Ad5FB4 vector particles for 90 min at 4°C, a temperature which allows cell attachment of the vector but blocks its endocytosis. Cells were then transferred to 37°C to induce the vector internalization, and the status of B7.1 and B7-H1 molecules at the cell surface examined by flow cytometry at 10-15 min after transfer. No modification of the B7.1 signal was detected upon Ad5FB4 endocytosis, even at high vector doses (10,000 vp/cell). By contrast, a discrete but significant decrease was observed in the levels of B7-H1 protein at the cell surface upon Ad5FB4 uptake, and in a vector dose-dependent manner: 15-17% at 5,000 vp/cell, and 22-25% at 10,000 vp/cell (Figure 8). However, the possibility existed that cell-bound vector particles masked the epitope of the B7-H1 molecules recognized by the specific antibody used in flow cytometry, and biased the results. To address this issue, cells were analyzed by flow cytometry immediately after incubation with Ad5FB4 at 4°C. The immunoreactivity of B7 molecules was found to be similar at the surface of control cells without Ad5FB4 and cells incubated with Ad5FB4 at 4°C (Figure 8, compare open bars and grey bars). The data clearly showed that the B7-H1 epitope was still accessible after Ad5FB4-cell binding, and suggested a cointernalization of B7-H1 molecules with Ad5FB4 particles. This supported the above-mentioned hypothesis that B7-H1 would be involved in Ad5FB4 endocytosis. In order to further dissect the mechanism of cellular entry of the Ad5FB4 vector and the contribution of the B7 molecules in this pathway, interactions between B7.1, B7-H1 and the adenoviral penton capsomeres were analyzed *in situ *within the context of live cell plasma membrane, using a recently developed enhancement of the bioluminescence resonance energy transfer (BRET) technique [29].

### Fluorescence microscopic analysis of B7.1, B7-H1 and Ad5FB4 interactions in situ

#### (i) Functionality of Rluc8- and YPet-fused B7-H1 and B7.1 molecules

B7.1 and B7-H1 proteins were fused to the Renilla luciferase variant Rluc8 as the energy donor [38], or to the YFP variant YPet (yellow fluorescent protein for energy transfer) as the energy acceptor [39]. The functionality of the resulting fusion proteins was monitored by their ability to function as alternative adenovirus receptors when overexpressed in HeLa cells, which were otherwise refractory to Ad5FB4. We found that HeLa cells cotransfected with plasmids expressing B7.1-Rluc8 and B7-H1-YPet (Figure 6e), or alternatively B7.1-YPet and B7-H1-Rluc8 (Figure 6f), were transduced by Ad5FB4 with the same efficiency as HeLa cells coexpressing non-fused B7-H1 and B7.1 proteins (Figure 6d).

#### (ii) Interactions between B7.1 and B7-H1

**Figure 9:**
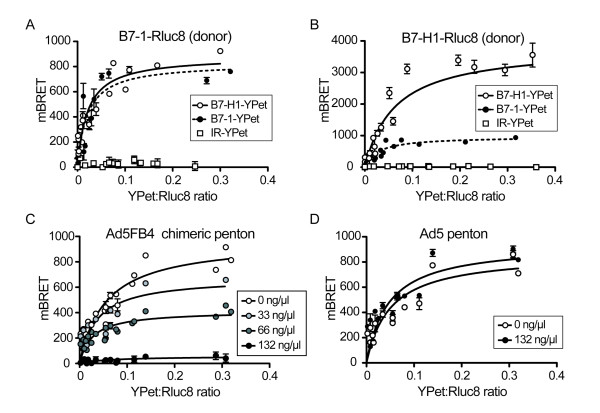
**BRET analysis of B7.1, B7-H1 and Ad5FB4 interactions *in situ***. **(A, B)**, ***Interactions between B7.1 and B7-H1 molecules***. Homodimeric and heterodimeric interactions were monitored in transfected HeLa cells using BRET donor saturation assays. Both B7.1 and B7-H1 were fused to Rluc8 to act as energy donors, alternatively; B7.1 (black circle), B7-H1 (open circle), and IR (open square) were fused to YPet to act as energy acceptors. Cells were cotransfected with plasmids expressing B7.1-Rluc8 or B7-H1-Rluc8 at constant dose, and with different doses of the other YPet-fused interactors. Results, expressed as mean milliBRET values (mBRET), were plotted versus the values of the YPet-to-Rluc8 ratio. **(C, D), *Effect of penton-HeLa cell interaction on B7.1-B7-H1 heterodimers***. B7.1-RLuc8 and B7-H1-YPet were coexpressed in HeLa cells and the BRET donor saturation curves were determined in the absence (basal BRET; open circle) or presence of increasing doses of penton protein, at 33 (light grey circle), 66 (dark grey circle), and 132 ng/μL (black circle), respectively. **(C)**, Ad5FB4 penton; **(D)**, Ad5 penton. Results, expressed as mBRET, were plotted versus the values of the YPet-to-Rluc8 ratio. Each point of the curves represented the average of six independent experiments (m ± SEM).

The interactions of B7.1 and B7-H1 with themselves and with each other were then analyzed by BRET, using B7-H1 and B7.1 fusion proteins coexpressed in HeLa cells. Donor saturation curves using B7.1 as donor (B7.1-Rluc8) and B7.1 as acceptor (B7.1-YPet) showed a direct interaction between B7.1 molecules (Figure 9A), confirming the occurrence of B7.1 homodimers at the cell surface, as previously described [40,41]. Likewise, the saturation curve of B7-H1 used as donor (B7-H1-Rluc8) and B7-H1 used as acceptor (B7-H1-YPet), confirmed the occurrence of B7-H1 homodimers (Figure 9B), as already reported [41]. When B7.1-Rluc8 and B7-H1-YPet were coexpressed as donor and acceptor, respectively (Figure 9A), and in the opposite configuration of B7-H1-Rluc8 with B7.1-YPet (Figure 9B), the two curves of BRET response superimposed and plateaued at the same value (ca. 800 mBRET), demonstrating the formation of B7.1/B7-H1 heterodimers. As a negative control, the cell surface-expressed insulin receptor (IR) fused to YPet (IR-YPet) and previously studied by BRET [42] did not show any detectable interaction with B7.1 or B7-H1, both used as the donor fusion proteins (Figure 9A, B).

#### (iii) Effect of Ad5FB4 on B7.1-B7-H1 interaction

We then applied the BRET analysis to explore the molecular events implicated in, or resulting from, the binding of Ad5FB4 to B7-H1/B7.1-expressing cells. When HeLa cells coexpressing donor B7.1-Rluc8 and acceptor B7-H1-YPet, as in the configuration of Figure 9A, were incubated with increasing doses of Ad5FB4 vector particles, a modest effect in BRET signals was observed at the highest dose of 10,000 vp/cell (not shown). We then used Ad5FB4 penton protein, the components of the adenoviral capsid responsible for cell attachment and endocytosis [17,18]. The rationale for the use of penton capsomeres instead of vector particles in BRET analysis was based on the following arguments: (i) like adenovirus particles, penton capsomeres are capable of cell attachment and entry [43-47]; (ii) in terms of macromolecules, isolated capsomeres have a higher solubility and dispersity compared to virus particles, of which suspensions are prone to aggregate; (iii) there are 12 pentons per adenoviral capsid, which represent ca. 5% of the total protein content of the virion. Considering that 3.4 × 10^12 ^adenovirus particles correspond to 1 mg protein [48], a solution of penton protein at the concentration of 132 ng/μL (the maximum concentration used in our dose-dependent curves) would correspond to a theoretical number of 9 × 10^9 ^vp/μL, viz. as many as 45,000 vp/cell in our standard BRET assays. Such high doses might provoke nondesired cytotoxic effects interfering with the metabolism of the B7 proteins, and justified the use of penton protein as opposed to vector particles. HeLa cells coexpressing donor B7.1-Rluc8 and acceptor B7-H1-YPet were incubated with increasing doses of Ad5FB4 penton protein, with Ad5 penton used as the control. Donor saturation curves showed an Ad5FB4 penton-dependent, dose-response decrease of the BRET signal (Figure 9C), whereas control Ad5 penton did not induce any change in the BRET signal, even at the maximum concentration of 132 ng/μL (Figure 9D).

The BRET data indicated that less B7.1/B7-H1 heterodimers were present at the cell surface after contact with Ad5FB4 penton, which suggested that the binding of Ad5FB4 penton to B7.1 prevented the formation of the B7.1/B7-H1 heterodimeric complexes, or/and that the complexes dissociated upon Ad5FB4 penton interaction. However, we could not exclude another mechanism, which consisted of a conformational change within the B7.1/B7-H1 complex upon Ad5FB4 penton binding. This structural modification might result in (i) a reorientation of the two partner proteins less favorable to generate a BRET signal, or/and (ii) in an increased distance between the donor and acceptor moieties of the two fusion proteins, without a complete dissociation of the complex [36]. Whatever the molecular mechanism, our BRET analysis demonstrated that a significant modification occurred in the B7.1-B7-H1 interaction or/and in their three-dimensional conformation and respective topology within the heterodimeric complex, upon Ad5FB4 penton interaction with the cell surface.

## Discussion

**Figure 10:**
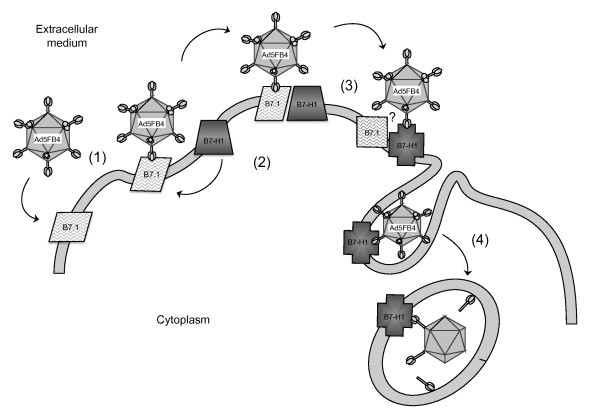
**Hypothetical model for the cell entry pathway of Ad5FB4**. ***Step 1***: binding of Ad5FB4 to B7.1 monomer. ***Step 2***: high affinity interaction between B7-H1 and B7.1, and formation of a B7.1-B7-H1 heterodimer. ***Step 3***: conformational changes in B7.1 or/and B7-H1, and possible modification of Ad5FB4-B7.1 interactions (question mark, ?). ***Step 4***: B7-H1-mediated endocytosis and internalization of Ad5FB4. The data obtained *in vitro *(SPR) and *in vivo *(cell binding assays and RNA interference) support the mechanisms proposed for steps 1 and 2; the molecular events depicted in step 3 are based on BRET analysis; the endocytic step 4 is supported by data of flow cytometry.

The interaction of adenovirus with host cells leading to a productive infection and to viral progeny represents a complex, multifactorial process which depends on both viral and cellular functions. The cell permissiveness to the virus is the result of a fine balance between intrinsic and extrinsic factors, but the efficacy of the primary event of virus-cell attachment is one major parameter which conditions the subsequent steps, and notably the cell entry of the virus and the transcription of its genome. In the present study, we showed that the efficiency of cell transduction by the chimeric fiber-pseudotyped Ad5FB4 vector depended on the coexpression of two molecules of the B7 family, B7-H1 and B7.1, at the surface of cells otherwise refractory or poorly permissive to Ad5FB4. This was observed in both human cells and murine dormant leukemia cells. Ad5FB4 efficiently bound to and transduced DA1-3b/d365 leukemia cells, a murine cell line derived from long-term dormant leukemia cells which overexpressed B7-H1/B7.1 molecules. Our data suggested that B7.1 was involved in Ad5FB4-cell attachment, but both B7-H1 and B7.1 were required for cell entry and efficient Ad5FB4-mediated transduction. BRET experiments demonstrated that B7-H1 and B7.1 formed homodimers and also heterodimers at the cell surface, and that B7-H1/B7.1 heterodimeric complex formation was altered upon cell interaction with Ad5FB4 penton capsomeres, the capsid components which are responsible for the steps of vector-cell attachment and entry. A tentative model for the cell attachment and entry pathway of Ad5FB4 is presented in Figure 10.

Under steady-state conditions, B7.1 is present as a heterogenous population of monomers and noncovalent dimers at the cell surface, and the same pattern has been described for B7-H1 [40,41]. However, interaction between B7-H1 and B7.1 molecules has also been observed [41]. B7.1 has a relatively low affinity for B7-H1 with, an intermediate affinity for CD28 and CTLA-4, and a high affinity for PD-1. Using T-cells deficient for different combinations of PD-1, B7.1, CD28 and CTLA-4, Butte *et al. *found a bidirectional inhibitory interaction between B7-H1 and B7.1 [6], with some overlapping of the binding domains of B7.1 and PD-1 on the B7-H1 molecule [6,9]. In the present study, we demonstrated and confirmed the occurrence of B7-H1/B7.1 heterodimeric complexes in live cells.

Targeting the molecules of the B7 family is potentially a promising strategy for cancer therapy for the following reasons. (i) Dormant tumor cells have developed several *in vivo *mechanisms to ensure their long-term persistence in their hosts (reviewed in [13]). One of these mechanisms is the overexpression of B7-H1 and B7.1, which shields these cells from CTLs [10-12]. This suggested that the equilibrium between minimal residual disease and host immune response could be modified to prevent disease recurrence. (ii) B7-H1 is frequently observed in human cancers and has a prognostic role for renal cell carcinoma [49]. (iii) B7-H1 and B7.1 play a role in immunoevasion through their expression in dendritic cells present in tumor draining lymph nodes. The delivery of various transgenes that may antagonize immunoevasion mechanisms, such as the chemokine CXCL10, which activates NK cells to kill B7-H1-overexpressing dormant leukemia cells [11], could help the host in the clearance of the cancer cells.

In this context, our finding that Ad5FB4 acted as a ligand of B7.1 monomer, and that Ad5FB4 penton negatively interferred with B7.1/B7-H1 heterodimer formation, made Ad5FB4 a unique adenoviral vector for cancer gene therapy, as it targeted cell surface molecules involved in the immunoevasion mechanisms. In the light of our observation in BRET analysis using Ad5FB4 penton protein, one could envisage to antagonize immunoevasion mechanisms by using Ad5FB4 penton as monovalent, single capsomeres, or as multivalent, double chimeric dodecamers (or dodecahedrons) formed by twelve penton base subunits of serotype 3 adenovirus (Ad3Dd) linked to twelve chimeric FB4 fibers (Ad3DdFB4). Ad3 dodecahedrons have been used as efficient protein vectors to bind to and enter mammalian cells [43-46]. It would be expected that Ad3DdFB4 would impair the formation of B7-H1/B7.1 heterodimers in tumor cells, induce their dissociation, or would cointernalize with one or the other molecule. The lower expression of immunoevasion complexes at the cell surface would in turn confer to tumor cells a higher susceptibility to tumor-reactive T-cells. Further characterization of the interaction between chimeric Ad3DdFB4 and B7-H1/B7.1 molecules *in vitro *and *in vivo *will be necessary to optimize the conditions for potential applications to cancer therapy.

## Conclusions

Tumor cells express specific molecules at their surface which may negatively affect their recognition by the immune system. This is the case for proteins of the B7 family, which play important roles in the immunoevasion of tumor cells. In the present study, we showed that leukemia cells DA1-3b/d365 derived from long-term dormant tumors, which are refractory to conventional adenovirus serotype 5 (Ad5)-based vectors, were permissive to Ad5FB4, an adenoviral vector carrying chimeric fibers. We found that the permissiveness of DA1-3b/d365 cells to Ad5FB4 correlated with the level of expression of B7.1 and B7-H1 molecules at their surface, and that the permissivity to Ad5FB4 could be reverted by RNA silencing of one or the other B7 gene transcript. Results from *in vitro *and *in vivo *experiments suggested that B7.1 and B7-H1 molecules played different roles in Ad5FB4-mediated transduction of DA1-3b/d365 cells, with B7.1 involved in Ad5FB4-cell attachment, and B7-H1 in Ad5FB4 internalization. We showed that the interaction between B7.1 and Ad5FB4 was mediated by the penton protein, the capsid component carrying the fiber projection. *In situ *BRET analysis showed that B7.1 and B7-H1 form heterodimeric complexes at the cell surface, and that Ad5FB4 penton capsomeres interfered negatively with the formation of B7.1/B7-H1 heterodimers. Our observation that the adenoviral vector Ad5FB4 interacted with cell surface molecules of the B7 family known to be implicated in immunoevasion mechanisms offers novel opportunities for cancer therapy using B7-H1/B7.1 heterodimers as cell surface targets, and Ad5FB4 vectors or Ad5FB4 penton capsomeres as therapeutic agents. Two different strategies might be envisaged: (i) naturally B7.1-targeted Ad5FB4 vectors can be designed for transferring therapeutic genes to B7.1/B7-H1-overexpressing cells, (ii) whereas soluble Ad5FB4 penton capsomeres would act via their negative interference with the B7-H1/B7.1 heterodimer formation.

## Competing interests

The authors declare that they have no competing interests.

## Authors' contributions

EG, KL, and SR carried out the molecular genetic and cell transduction studies, CC carried out the BRET studies, and PF the SPR analyses. SSH purified the adenovirus capsid proteins. PF and SSH participated in the design of the study and the discussion of the experimental data. JCD, BQ and MC conceived of the study, and participated in its design and coordination. PB and MC wrote the manuscript. All authors read and approved the final manuscript.
